# Incomplete Downregulation of CD4 Expression Affects HIV-1 Env Conformation and Antibody-Dependent Cellular Cytotoxicity Responses

**DOI:** 10.1128/JVI.00484-18

**Published:** 2018-06-13

**Authors:** Jérémie Prévost, Jonathan Richard, Halima Medjahed, Audrey Alexander, Jennifer Jones, John C. Kappes, Christina Ochsenbauer, Andrés Finzi

**Affiliations:** a Centre de Recherche du CHUM, Montreal, QC, Canada; b Department of Microbiology, Infectious Diseases and Immunology, Université de Montréal, Montreal, QC, Canada; c Department of Medicine, University of Alabama at Birmingham, Birmingham, AL, USA; d Department of Microbiology and Immunology, McGill University, Montreal, QC, Canada; Icahn School of Medicine at Mount Sinai

**Keywords:** HIV-1, Nef, CD4, Env, gp120, ADCC, NKG2D, luciferase, IMC, A32, CD4i

## Abstract

HIV-1-infected cells expressing envelope glycoproteins (Env) in the CD4-bound conformation on their surfaces are targeted by antibody-dependent cellular cytotoxicity (ADCC) mediated by CD4-induced (CD4i) antibodies and sera from HIV-1-infected individuals (HIV^+^ sera). By downregulating the surface expression of CD4, Nef prevents Env-CD4 interaction, thus protecting HIV-1-infected cells from ADCC. HIV-1 infectious molecular clones (IMCs) are widely used to measure ADCC. In order to facilitate the identification of infected cells and high-throughput ADCC analysis, reporter genes (e.g., the Renilla luciferase [LucR] gene) are often introduced into IMC constructs. We evaluated the susceptibility of HIV-1-infected CD4^+^ T lymphocytes to ADCC using a panel of parental IMCs and derivatives that expressed the LucR reporter gene, utilizing different molecular strategies, including one specifically designed to retain Nef expression. We found that in some of these constructs, Nef expression in CD4^+^ T cells was suboptimal, and consequently, CD4 downregulation was incomplete. CD4 molecules remaining on the cell surface resulted in the exposure of ADCC-mediating CD4i epitopes on Env and a dramatic increase in the susceptibility of the infected cells to ADCC. Strikingly, protection from ADCC was observed when cells were infected with the parental IMC, which exhibited strong CD4 downregulation. This discrepancy between the parental and Nef-impaired viruses was independent of the strains of Env expressed, but rather, it was correlated with the levels of CD4 surface expression. Overall, our results indicate that caution should be taken when selecting IMCs for ADCC measurements and that CD4 downregulation needs to be carefully monitored when drawing conclusions about the nature and magnitude of ADCC.

**IMPORTANCE** In-depth understanding of the susceptibility of HIV-1-infected cells to ADCC might help establish correlates of vaccine protection and guide the development of HIV-1 vaccine strategies. Different ADCC assays have been developed, including those using infectious molecular clones (IMCs) carrying a LucR reporter gene that greatly facilitates large-scale quantitative analysis. We previously reported different molecular strategies for introducing LucR while maintaining Nef expression and function and, consequently, CD4 surface downregulation. Here, we demonstrate that utilizing IMCs that exhibit impaired Nef expression can have undesirable consequences due to incomplete CD4 downregulation. CD4 molecules remaining on the cell surface resulted in the exposure of ADCC-mediating CD4i epitopes on Env and a dramatic increase in the susceptibility of the infected cells to ADCC. Overall, our results indicate that CD4 downregulation needs to be carefully monitored when drawing conclusions about the nature and magnitude of ADCC.

## INTRODUCTION

Recent efforts aimed at understanding antibody-dependent cellular cytotoxicity (ADCC) against HIV-1-infected cells uncovered several strategies put in place by the virus to limit exposure of vulnerable CD4-induced (CD4i) epitopes. Interaction of Env with the CD4 receptor was reported to be critical for exposing epitopes recognized by ADCC-mediating antibodies (Abs) (1–3). HIV-1 achieves protection from ADCC by limiting Env-CD4 interaction by downregulating CD4 and preventing Env accumulation at the surface of infected cells (1, 3–7). Two accessory proteins, Nef and Vpu, impact ADCC sensitivity via reduction of cell surface expression of CD4 (1, 3), while Env accumulation is tightly controlled through efficient internalization (6) and Vpu-mediated BST-2 downregulation (4, 5, 7). Therefore, these accessory proteins protect HIV-1-infected cells from ADCC mediated by CD4i nonneutralizing Abs (nnAbs). Thus, cells infected with primary viruses coding for functional Nef and Vpu proteins are largely resistant to ADCC induced by these nnAbs (3, 7–13). These findings are in agreement with structural information indicating that ADCC-mediating nnAbs target a highly conserved region in the gp120 inner domain that is buried inside the untriggered Env trimer and becomes exposed only upon CD4 engagement (2, 3, 8, 14–17) Thus, it is to be expected that in the presence of functional Vpu and Nef, HIV-1-infected cells will be largely refractory to CD4i nnAb-mediated ADCC. Several studies, however, have reported ADCC activity by such antibodies against HIV-1-infected cells (18–33), suggesting the CD4i epitopes were accessible. Since all of these studies used HIV-1 infectious molecular clones (IMCs) carrying the Renilla luciferase (LucR) reporter gene for sensitive quantification of infection and infection inhibition, we wondered whether the molecular design of the LucR reporter IMC might have impaired Nef functions that impacted the conformation sampled by Env at the surface of infected cells. Of note, the original reporter IMC strategy encompassed an isogenic proviral backbone from which heterologous *env* strains could be expressed in *cis* and encoding LucR in frame with a T2A “ribosome-skipping” peptide intended to drive Nef expression (referred to as Env-IMC-LucR.T2A) (34). Supporting the possibility of Env conformation being impacted, we previously reported that the strategy used to create LucR reporter IMCs can affect Nef expression on CD4^+^ T cells (35), which in turn may affect the Env conformation due to incomplete CD4 downregulation (1, 3, 13, 36). Of note, we found that a revised molecular strategy utilizing a modified encephalomyocarditis virus (EMCV) internal ribosome entry site (IRES) element in lieu of T2A (Env-IMC-LucR.6ATRi) can normalize Nef expression and function compared to the Env-IMC-LucR.T2A molecular strategy (35, 37). In order to test this possibility, we evaluated the CD4i nnAb binding and ADCC susceptibility of primary CD4^+^ T cells infected with panels of parental IMCs and LucR-reporter IMC derivatives in which different strategies to drive Nef expression were applied. Cells infected with certain LucR viruses that exhibited physiological levels of Nef expression downregulated surface CD4 efficiently, though not always completely. Consequently, their levels of CD4i nnAb binding were similar to that of cells infected with the parental viruses, as were their levels of protection against ADCC by CD4i antibodies. However, importantly, we found that in some IMC-LucR constructs, Nef expression in CD4^+^ T cells was suboptimal, and consequently, CD4 downregulation from the cell surface was less efficient. This allowed Env-CD4 engagement and the exposure of CD4i epitopes otherwise occluded on cells infected with parental IMCs. Consequently, while cells infected with parental IMCs presented robust CD4 downregulation and were protected from ADCC mediated by the antibody A32 and HIV^+^ sera, cells infected with the LucR reporter IMCs that displayed impaired Nef expression (i.e., encoding LucR.T2A) were highly susceptible to ADCC mediated by these ligands.

## RESULTS

### Molecular strategies for Nef expression in reporter HIV-1 affect CD4 downregulation.

**FIG 1:**
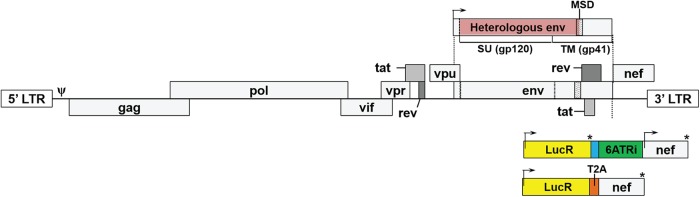
Schematic representation of HIV-1 IMC, Env-IMC, and their LucR reporter virus derivatives. Shown is the HIV-1 proviral genome representing either a full-length IMC (e.g., T/F strain) or a pNL4.3-based isogenic backbone engineered to encode the Env ectodomain (i.e., SU/gp120 and the extracellular and part of the membrane-spanning domain [MSD] portion of TM/gp41; shaded in pink) of heterologous HIV-1 strains. The cytoplasmic tail of Env is derived from backbone strain NL4-3, as is the signal peptide sequence. Also shown is how IMCs were modified for reporter gene expression by the insertion into the IMC backbone of the Renilla luciferase (LucR) open reading frame (yellow), followed by either the previously described 26-nucleotide (nt) IRES spacer region (blue) and the modified EMCV IRES element, 6ATRi (green), which drives expression of wild-type Nef; a 3-amino-acid-long functional deletion mutant (not shown); or the T2A ribosome-skipping peptide sequence (orange) in frame with *nef*. The arrows indicate translation start sites. *, stop codon. The molecular strategies are based on those we previously described (34, 35). LTR, long terminal repeat; Ψ, Psi packaging element.

**FIG 2:**
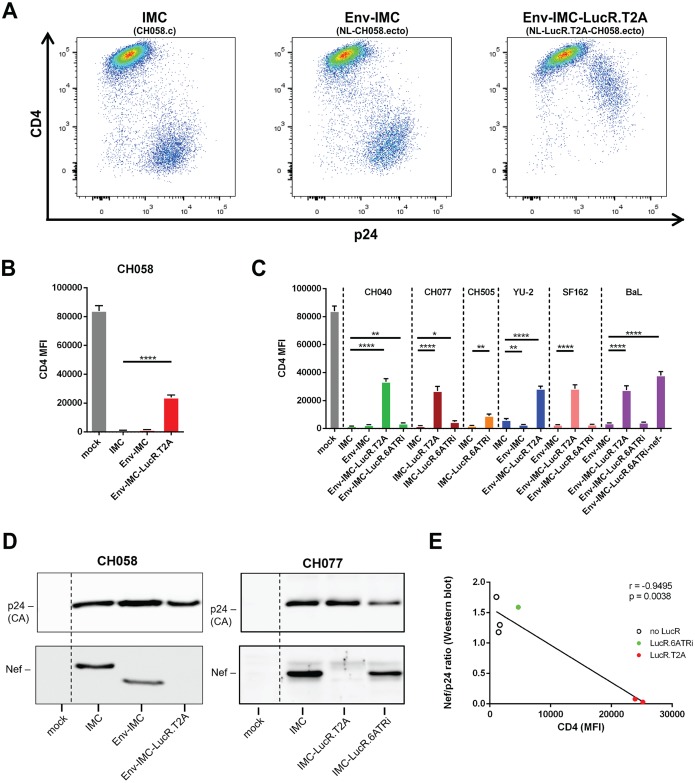
Impact of the molecular strategy for LucR element insertion on Nef expression from reporter HIV-1 and CD4 downregulation. Cell surface staining with anti-CD4 antibody OKT4 of primary CD4^+^ T cells either mock infected or infected with IMCs expressing different Env strains (CH058, CH040, CH077, CH0505, YU2, SF162, and BaL) and expressing or not the LucR reporter. (A to C) Histograms depicting representative CD4 stainings (A) and the MFI of the infected (p24^+^) population obtained in at least 5 independent experiments (B and C). (D) Nef expression from cells infected with the indicated parental and LucR IMCs encoding CH058 or CH077 Env was monitored by Western blotting with antibodies directed against CA/p24 (for normalization) and Nef. (E) Nef expression levels correlated with detection of CD4 at the surfaces of infected (p24^+^) cells using a Pearson correlation test. The data are shown as means and standard errors of the mean (SEM). Statistical significance was tested using an unpaired *t* test (*, *P* < 0.05; **, *P* < 0.01; ****, *P* < 0.0001).

HIV-1 IMCs carrying panels of heterologous HIV-1 *env* sequences (Env-IMCs) in an isogenic backbone and expressing the Renilla luciferase (LucR) reporter, which allows highly sensitive, quantitative readout of productive infection (34), have been widely used to evaluate the susceptibility of HIV-1-infected cells to ADCC mediated by vaccine-elicited antibodies and several CD4i nnAbs (18–33). However, we previously reported that some of these LucR reporter IMCs, particularly those utilizing a ribosome-skipping T2A peptide strategy to link Renilla luciferase with Nef (Env-IMC-LucR.T2A), were unable to fully downregulate CD4 due to impaired Nef expression in CD4^+^ T cells (35). In a modified molecular approach, we introduced a bicistronic LucR.IRES-*nef* cassette in lieu of LucR.T2A-*nef* by utilizing the EMCV-modified 6ATR IRES element (6ATRi) to drive *nef* expression (Env-IMC-LucR.6ATRi) and showed that it can normalize Nef expression and function compared to the T2A molecular strategy (35, 37). Figure 1 schematically illustrates these molecular approaches. In this study, we aimed to elucidate whether different strategies to drive Nef expression affect the sensitivity of reporter HIV-1 IMCs to ADCC compared to their parental nonreporter IMCs. To this end, we first compared the abilities of a panel of IMCs (described in Materials and Methods) to downregulate CD4. In addition to previously reported replication-competent Env-IMC and Env-IMC-LucR.T2A viruses, we included an expanded panel of Env-IMC-LucR.6ATRi viruses carrying further transmitted/founder (T/F) and reference *env* strains, as well as novel full-length T/F IMCs into which the LucR.6ATRi cassette was inserted. In order to evaluate the abilities of the different parental and LucR IMCs to downregulate CD4 from the cell surface, primary CD4^+^ T cells from HIV-1-uninfected individuals were infected as described in Materials and Methods and 48 h later incubated with the anti-CD4 antibody OKT4. The infected cells were identified by intracellular p24 staining and analyzed by flow cytometry as indicated in Materials and Methods. To help visualize the impact that the LucR.T2A cloning strategy had on CD4 downregulation, representative flow cytometry dot plots are provided in Fig. 2A. Cells infected (p24^+^) with the parental CH058.c T/F virus exhibited robust CD4 downregulation (Fig. 2A, left). This was recapitulated with a pNL4.3-based Env-IMC expressing the CH058 Env (Fig. 2A, center). However, in the strain-matched Env-IMC-LucR.T2A virus (NL-LucR.T2A-B.CH058.ecto), CD4 downregulation was dramatically reduced (Fig. 2A and B). This finding was consistent with what we previously described for the BaL *env*-bearing counterpart, NL-LucR.T2A-BaL.ecto, and matched the Nef-minus control (Fig. 2C) (35). The same impairment of CD4 downregulation compared to parental Env-IMC was observed with additional Env-IMC-LucR.T2A viruses expressing different T/F and reference strain Envs (CH040, YU2, SF162, and BaL), indicating that the observed effect was independent of the Env being expressed (Fig. 2C). Notably, the T2A strategy to drive Nef expression also adversely affected CD4 downregulation when LucR.T2A was introduced into the parental CH077.t T/F IMC (Fig. 2C), indicating that the lack of functional Nef expression and CD4 downregulation is not exclusive to the laboratory-adapted pNL4.3 backbone. Interestingly, introduction of the LucR.6ATRi element into two T/F IMCs (CH077.t and CH0505s) or several Env-IMC proviruses allowed downregulation of CD4 to physiological levels, similar to their parental counterparts, but not always as efficiently (Fig. 2C, compare CH040, CH077, and CH505). As expected, and in agreement with our previous publication (35), impaired CD4 downregulation was strongly correlated with deficient Nef expression in T cells (Fig. 2D and E) and with deficient Nef-mediated major histocompatibility complex class I (MHC-I) downregulation (not shown).

### CD4 downregulation affects Env conformation and ADCC responses.

**FIG 3:**
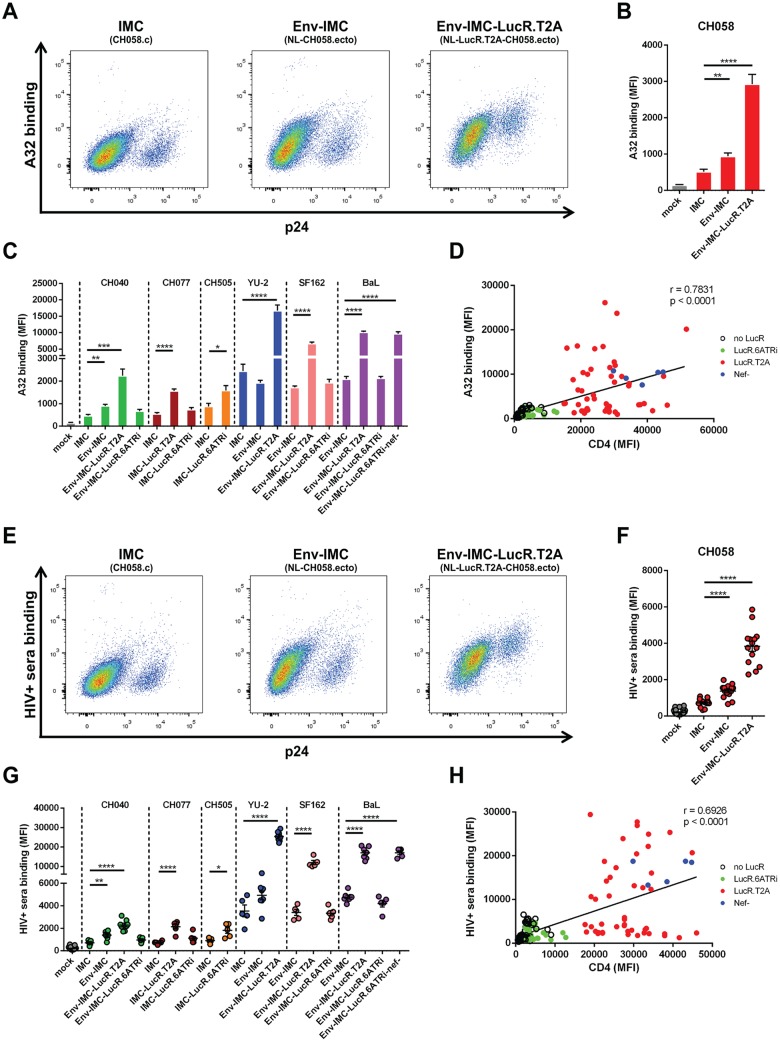
Impact of the molecular strategy for LucR reporter element insertion on Env conformation. Cell surface staining of primary CD4^+^ T cells either mock infected or infected with IMCs expressing different Env strains (CH58, CH40, CH77, CH505, YU2, SF162, and BaL) and expressing or not the LucR reporter gene with A32 (A to D) or HIV^+^ sera (E to H). (A to C and E to G) Histograms depicting representative A32 or HIV^+^ serum staining (A and E) and the MFI in the infected (p24^+^) population (B, C, F, and G) obtained in at least 5 independent experiments. (D and H) Spearman rank correlations between the levels of cell surface CD4 (detected with the anti-CD4 Ab OKT4) and Env staining performed with A32 or HIV^+^ sera using different IMC constructs. The data are shown as means and SEM. Statistical significance was tested using an unpaired *t* test (B, C, F, and G) or a Spearman correlation test (D and H) (*, *P* < 0.05; **, *P* < 0.01; ***, *P* < 0.001; ****, *P* < 0.0001).

We previously established that inefficient CD4 downregulation results in Env-CD4 interaction that causes exposure of Env CD4i epitopes on the surfaces of infected cells (1, 3, 13). Nevertheless, Nef-impaired Env-IMC-LucR.T2A viruses have been used to asses CD4i nnAb-mediated ADCC *in vitro*, while Env strain-matched reporter viruses utilizing the 6ATRi element to drive Nef expression had not yet been investigated for ADCC assays. We therefore investigated whether the impaired CD4 downregulation observed for some LucR reporter IMCs (Fig. 2A to C) was sufficient to alter Env conformation. Primary CD4^+^ T cells were infected with the same panels of viruses described above. Exposure of CD4i epitopes was monitored with the nnAb antibody, A32, which recognizes an anti-cluster A epitope normally occluded in the untriggered trimer (2, 3, 8, 14–17), as well as with sera from HIV-1-infected individuals (HIV^+^ sera). Dramatic differences in Env recognition by A32 (Fig. 3A to C) and HIV^+^ sera (Fig. 3E to G) were observed among the different panels of IMCs expressing the same Env. For example, cells infected with the wild-type CH058.c T/F IMC or its cognate nonreporter Env-IMC were poorly recognized by these ligands (Fig. 3A and E), but when the same Env was expressed in the context of the Env-IMC-LucR.T2A backbone, Env was much more readily recognized by the ligands (mean fluorescence intensities [MFI] were 3- to 5-fold higher) (Fig. 3B and F). Varying degrees of significant increases in Ab binding were also seen for the other T2A-encoding IMCs compared to their Env strain-matched parental viruses and were consistent with the extent of Nef-deficient virus (Fig. 3C and G, panel of BaL Env-expressing IMCs). With regard to Ab binding, LucR.6ATRi-containing reporter IMCs closely resembled parental viruses (Fig. 3C and G). Accordingly, highly significant correlations (*P* < 0.0001) were established between the amounts of CD4 detected on the cell surface and Env recognition by A32 (Fig. 3D) and HIV^+^ sera (Fig. 3H).

**FIG 4:**
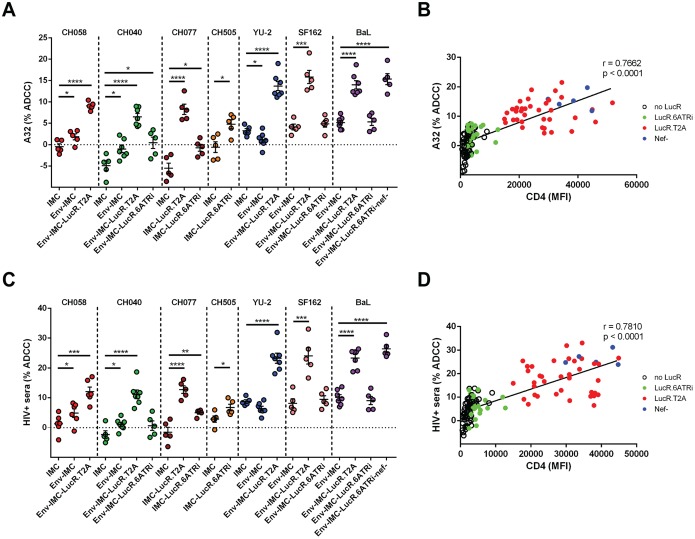
Susceptibility of cells infected with different parental and reporter IMCs to ADCC mediated by A32 and HIV^+^ sera. Primary CD4^+^ T cells infected with IMCs expressing different Env strains (CH58, CH40, CH77, CH505, YU2, SF162, and BaL) and expressing or not the LucR reporter gene were used as target cells, and autologous PBMCs were used as effector cells in a well-established FACS-based ADCC assay (1, 9, 10, 60, 84). (A and C) Percentages of ADCC-mediated killing obtained with A32 or HIV^+^ sera from 5 HIV-1-infected individuals obtained in at least 5 independent experiments. (B and D) Correlations between levels of cell surface CD4 and ADCC responses mediated by A32 or HIV^+^ sera, using the same panel of IMC constructs, detected with the FACS-based ADCC assay. Statistical significance was tested using an unpaired *t* test (A and C), a Pearson correlation test (B), or a Spearman rank correlation test (D) (*, *P* < 0.05; **, *P* < 0.01; ***, *P* < 0.001; ****, *P* < 0.0001).

Importantly, increased Env recognition translated into higher susceptibility to ADCC (Fig. 4). Cells infected with the parental IMC were completely (encoding T/F Env strains) or largely (encoding chronic and laboratory-adapted strains, YU-2, SF162, and BaL) resistant to ADCC mediated by these ligands, and LucR.6ATRi-encoding reporter counterparts displayed similar or slightly higher ADCC than the respective parental strain. In contrast, cells infected with viruses unable to efficiently downregulate CD4 were highly susceptible. Again, strong (*r* > 0.76) and highly significant (*P* < 0.0001) correlations were established between cell surface CD4 levels and ADCC mediated by A32 (Fig. 4B) and HIV^+^ sera (Fig. 4D). These results strongly advocate for careful characterization of the CD4 downregulation properties of any HIV-1 IMC, including reporter viruses, chosen and intended to measure ADCC, as any IMC with impaired Nef (or Vpu) function may have dramatic effects on Env conformation and thus introduce significant bias toward nonneutralizing CD4i Abs.

### Impaired downregulation of NKG2D ligands modulates ADCC responses.

**FIG 5:**
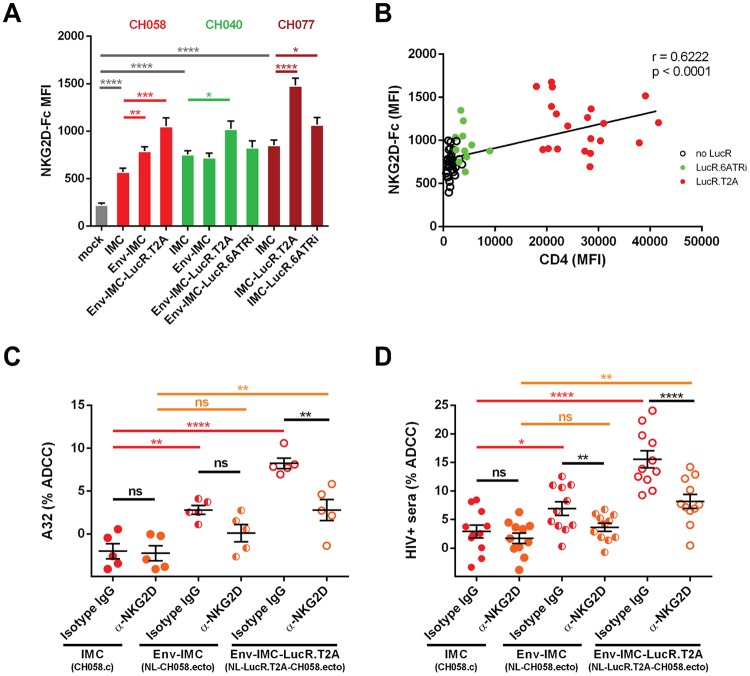
Effect of the molecular strategy for LucR reporter element insertion on the expression of NKG2D ligands. Shown are the results of cell surface staining of primary CD4^+^ T cells either mock infected or infected with IMC constructs expressing different Env strains (CH058, CH040, and CH077) and expressing or not the LucR reporter gene with an NKG2D-Fc chimera protein that binds to NKG2D ligands. (A) MFI in the infected (p24^+^) population obtained in at least 5 independent experiments. (B) Spearman rank correlation between CD4 levels and NKG2D ligand expression. (C and D) ADCC responses obtained in the presence of an anti-NKG2D Ab or its isotype control, as described in Materials and Methods. The data are shown as means and SEM. Statistical significance was tested using an unpaired *t* test (A), a Spearman correlation test (B), or an unpaired (C) or a paired (D) one-way analysis of variance (ANOVA) with a Holm-Sidak posttest (*, *P* < 0.05; **, *P* < 0.01; ***, *P* < 0.001; ****, *P* < 0.0001; ns, nonsignificant).

While antibody recognition of target cells is absolutely required for ADCC responses, additional interactions between target and effector cells have been shown to be necessary to activate effector cells, including NK cells. For example, NK cell effector functions are modulated by a balance between signals delivered through inhibitory (e.g., KIR and CD94/NKG2A), activating (e.g., CD16, NKG2D, DNAM-1, and NKp46), or coactivating (e.g., NTB-A and 2B4) receptors that either suppress or enhance NK cell activity (38). It has been shown that Nef decreases the expression of NKG2D ligands (MICA, ULBP1, and ULBP2) (39, 40), thus preventing their interaction with the NK cell-activating NKG2D receptor. Since the NKG2D receptor is known to modulate ADCC responses against HIV-1-infected cells (36, 41), we evaluated the capacities of IMCs expressing T/F Envs CH058, CH040, and CH077 to downregulate NKG2D ligands. To evaluate this, primary CD4^+^ T cells were isolated from non-HIV-infected individuals and infected with these IMCs as described above. The cultures were then stained with a recombinant human NKG2D-Fc chimera that recognizes several NKG2D ligands (41–44) or with a matched isotype control. Infected cells within these cultures were identified by intracellular p24 staining. As previously reported (45), HIV-1 infection enhanced NKG2D-Fc detection compared to mock-infected cells (Fig. 5A). This enhancement, however, was significantly greater when cells were infected with IMCs comprising the LucR.T2A element and was correlated with CD4 levels present at the cell surface (Fig. 5B). We next addressed whether the increased expression of NKG2D ligands enhanced susceptibility to ADCC mediated by A32 and HIV^+^ sera. As previously reported (1, 3, 8, 13, 36) and shown in Fig. 4, cells infected with the parental viruses were not sensitive to ADCC mediated by A32 (Fig. 5C) or antibodies within HIV^+^ sera (Fig. 5D), using autologous peripheral blood mononuclear cells (PBMCs) as effector cells. Supporting Nef's role in evading ADCC (1, 3, 8, 13, 36, 41), cells infected by IMCs with impaired Nef expression (i.e., LucR.T2A viruses) were more susceptible to ADCC mediated by either A32 (Fig. 5C) or HIV^+^ sera (Fig. 5D). In agreement with a role for NKG2D ligands in ADCC responses against HIV-1-infected cells (36, 41), addition of a blocking anti-NKG2D antibody, but not an isotype control, significantly decreased the susceptibility of (Env-)IMC LucR.T2A-infected cells to ADCC mediated by A32 and antibodies contained within HIV-1^+^ sera (Fig. 5C and D). Altogether, these results indicate that, in addition to the exposure of ADCC-mediating epitopes induced by the presence of CD4 at the cell surface, the accumulation of NKG2D-activating ligands promotes NK cell cytotoxicity for cells infected with IMCs unable to express physiological levels of Nef.

## DISCUSSION

**FIG 6:**
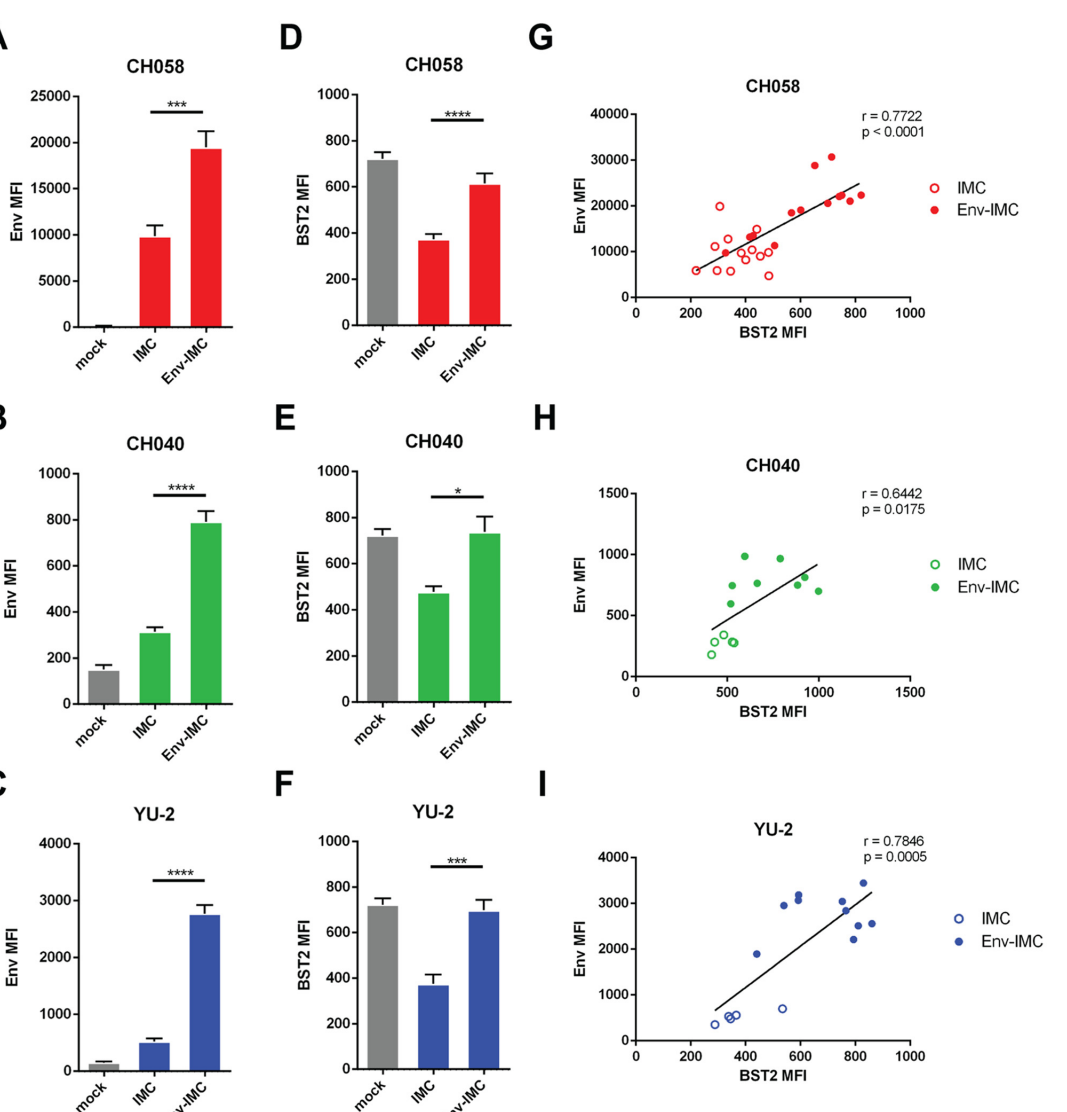
Impact of the proviral backbone on BST2 downregulation and Env detection at the cell surface. Shown are the results of cell surface staining of primary CD4^+^ T cells either mock infected or infected with IMC constructs expressing different Envs (CH58, CH40, and YU2) with the conformation-independent anti-Env 2G12 (A), 10e8 (B and C), or anti-BST2 (D to F). (A to F) MFI in the infected (p24^+^) population obtained in at least 5 independent experiments. (G to I) Correlations between the levels of cell surface BST2 and Env staining performed with 2G12 or 10e8 using different IMC constructs. The data are shown as means and SEM. Statistical significance was tested using an unpaired *t* test (A to F) or a Pearson correlation test (G to I) (*, *P* < 0.05; ***, *P* < 0.001; ****, *P* < 0.0001).

While ADCC responses against HIV-1 have been associated with protection against HIV-1 transmission and disease progression (23, 46–51), there are conflicting reports regarding the ability of nonneutralizing CD4i Abs to mediate ADCC. Consistent with the occluded nature of the epitopes recognized by these Abs, several groups have shown that cells infected with full-length HIV-1 are resistant to ADCC mediated by the Abs (3, 8–13). However, CD4i Abs have also been reported to mediate potent ADCC (18–23, 52–59). Part of this conundrum was recently explained by performing a side-by-side comparison of the different assays used to measure ADCC responses (60). It was shown that assays that do not differentiate virus-infected from uninfected cells (granzyme B and NK cell activation) or that rely on gp120-coated cells (rapid fluorometric antibody-dependent cellular cytotoxicity [RFADCC] assay) overestimate ADCC responses mediated by antibodies to CD4i epitopes. These assays are severely biased in favor of CD4i antibodies as a consequence of the coating of uninfected bystander cells by shed gp120 (10, 60). However, this did not explain why additional studies found strong ADCC of these Abs when measuring their activities against cells infected with full-length IMC LucR.T2A viruses (18–33), in which the readout was sensitive detection of a provirally encoded LucR reporter gene (i.e., avoiding measurement of bystander killing) and a molecular strategy utilizing a T2A ribosome-skipping peptide intended to provide Nef expression. Since increasing evidence supports a role of Env conformation in the susceptibility of HIV-1-infected cells to ADCC (61–64), we explored the possibility that Env conformation at the surface of cells infected with these reporter viruses might differ from those of the parental viruses due to altered interactions with CD4. We first evaluated the abilities of these viruses to downregulate the CD4 receptor in comparison with their parental IMCs. In agreement with our previous report (35), we found that the presence of the T2A peptide did not, as intended, support Nef expression in primary T cells, and consequently, CD4 downregulation was impaired. The remaining levels of CD4 at the surface of cells infected with these LucR.T2A reporter IMCs were sufficient to expose CD4i epitopes on Env, permitting recognition by A32 and HIV^+^ sera. An alternative molecular strategy to achieve LucR, as well as Nef, expression (6ATRi) largely, but not fully, resolved these deficiencies. In marked contrast to Nef-deficient viruses, cells infected with the parental IMCs (e.g., T/F IMC, CH058.c, CH040.c, CH077.t, and CH0505s) exhibited robust CD4 downregulation and thus did not expose the epitopes. Accordingly, cells infected with the parental viruses were resistant to ADCC mediated by these ligands (Fig. 4 and 5). In addition, we observed that IMCs with impaired Nef expression were unable to fully downregulate NKG2D ligands, thus contributing to the enhanced susceptibility of these cells to CD4i antibody-mediated ADCC responses (Fig. 5). For example, cells infected with CH058 Env-IMC were more susceptible to ADCC mediated by A32 and HIV^+^ sera than those infected with the parental virus (Fig. 4 and 5). This could be at least partially explained by the presence of higher levels of NKG2D ligands at the surfaces of CH058 Env-IMC-infected cells (Fig. 5A). Interestingly, we also found that some Env-IMCs, including CH058, failed to downregulate the restriction factor BST-2 to the same extent as the parental virus, resulting in increased Env levels at the cell surface (Fig. 6) and explaining the better recognition of these infected cells by A32 and HIV^+^ sera (Fig. 3B and F). Therefore, our results strongly advocate for carefully controlling for Nef expression and function, as well as BST-2 downregulation and Env expression, when selecting IMCs to measure ADCC.

The establishment of a large panel of viruses has proven extremely useful to standardize neutralization assessments of anti-Env antibodies and to evaluate their breadth and potency (65–67). Similar efforts are being considered for evaluating the breadth of ADCC responses against HIV-1-infected cells (68). One attractive strategy might be to generate large panels of reporter IMCs expressing Envs from circulating strains, which offers the advantage of highly sensitive, quantitative, and rapid assay readout, which avoids inadvertent measurement of bystander killing. The fact that this strategy worked well for neutralization assays (69–71) does not, however, guarantee that it will be the same for ADCC without undertaking further extensive optimization for ADCC assay-specific criteria. We believe that assays to measure Ab and effector cell interactions with infected cells are significantly more complex than those assessing Ab neutralization of viral particles because, in addition to Env expression and conformation, additional players, such as NKG2D ligands, can affect the readout of the assay. Therefore, our results strongly suggest that additional efforts should be devoted to identify and optimize reporter IMC strategies that ensure wild-type-like Nef expression and function before developing them into Env strain panels. We found LucR reporter IMCs encoding the modified EMCV IRES 6ATRi element expressed levels of Nef, and mediated CD4 downregulation, similarly to the cognate parental viruses. Consequently, Env conformation was less affected than with LucR IMCs utilizing the T2A peptide approach. Nevertheless, small but significant differences remained and varied depending on the 6ATRi-containing IMC that was used. For example, when 6ATRi was introduced in the pNL4.3 backbone coding for BaL Env, ADCC responses were similar to those obtained for the nonreporter pNL4.3. However, in the context of IMCs coding for CH040, CH077, and CH505 Envs, ADCC susceptibility was significantly higher than for their parental counterparts (Fig. 4). While small differences in CD4 and NKG2D ligand downregulation might partially explain these differences, additional parameters, including Env expression and Vpr-mediated upregulation of NKG2D ligands (45), could also affect ADCC responses and should be carefully evaluated. Our results highlight just some of the important elements of the intrinsic complexity in the interplay between Env and accessory proteins in modulating the susceptibility of infected cells to ADCC. A better comprehension of the susceptibility of HIV-1-infected cells to ADCC might help us better understand the correlates of vaccine protection and guide the development of HIV eradication strategies. Our results strongly advocate for carefully measuring parameters that may affect Env conformation and accessory protein function when measuring anti-HIV ADCC responses.

## MATERIALS AND METHODS

### Ethics statement.

Written informed consent was obtained from all study participants (the Montreal Primary HIV Infection Cohort [72, 73] and the Canadian Cohort of HIV Infected Slow Progressors [74–76]), and the research adhered to the ethical guidelines of Centre de Recherche du CHUM (CRCHUM) and was reviewed and approved by the CRCHUM Institutional Review Board (ethics committee approval number CE 16.164 -CA). The research adhered to the standards indicated by the Declaration of Helsinki. All the participants were adults and provided written informed consent prior to enrollment, in accordance with Institutional Review Board approval.

### Cell lines and isolation of primary cells.

HEK293T human embryonic kidney cells (obtained from the ATCC) were grown as previously described (3, 9). TZM-bl cells were cultured as we described previously (77). Primary human PBMCs and CD4^+^ T cells were isolated, activated, and cultured as previously described (3, 9). Briefly, PBMCs were obtained by leukapheresis, and CD4^+^ T lymphocytes were purified from resting PBMCs by negative selection using immunomagnetic beads (StemCell Technologies, Vancouver, BC, Canada) according to the manufacturer's instructions and were activated with phytohemagglutinin-L (10 μg/ml) for 48 h and then maintained in RPMI 1640 complete medium supplemented with recombinant interleukin 2 (rIL-2) (100 U/ml).

### Proviral constructs.

We previously reported the generation of proviral plasmids of IMCs of T/F clade B HIV-1 strains pCH040.c, pCH058.c, and pCH077.t (accession numbers JN944939, JN944940, and JN944941) (78), and that of the brain-derived HIV-1 strain YU-2 (79), in which *vpu* was corrected to yield pYU-2c, and the clade C T/F IMC pCH0505s (80) was also previously described. Proviral constructs, referred to collectively as Env-IMCs, comprising an HIV-1 NL4.3 (M19921.2)-based isogenic backbone engineered for the insertion of heterologous *env* strain sequences and expression in *cis* of full-length Env, were previously described (34). The proviral plasmids of replication-competent Env-IMCs utilized in this study are those encoding the Env ectodomain of T/F strains (pNL-B.CH040.ecto, pNL-B.CH058.ecto, pNL-CH077.ecto, and pNL-C.CH0505s.ecto) and reference strains (pNL-B.YU-2.ecto, pNL-B.SF162.ecto, and pNL-B.BaL.ecto). In the same study, we reported the construction of *env* strain-matched, replication-competent reporter virus derivatives of Env-IMCs that encode Renilla luciferase (LucR) followed in frame by a ribosome-skipping T2A peptide intended to drive Nef expression, collectively referred to as Env-IMC-LucR.T2A viruses (34). The proviral plasmids utilized here were pNL-LucR.T2A-B.CH040.ecto, pNL-LucR.T2A-B.CH058.ecto, pNL-LucR.T2A-CH077.ecto, pNL-LucR.T2A-B.YU-2.ecto, pNL-LucR.T2A-B.SF162.ecto, pNL-LucR.T2A-B.BaL.ecto, and pNL-LucR.T2A-B.Bal.ecto-Nef^stop^ (34).

In a modified molecular approach, we replaced the bicistronic LucR.T2A-*nef* fragment with a bicistronic LucR.IRES-*nef* cassettes utilizing the EMCV-modified 6ATR IRES element (6ATRi) to drive *nef* expression, resulting in Env-IMC-LucR.6ATRi viruses (35).

The LucR reporter virus derivatives of CH077.t and CH0505sT/F IMCs included in this study were constructed similarly to IMC-LucR.T2A (CH077.t-LucRT2A [81]) or IMC-LucR.6ATRi (CH077.t-LucR.6ATRi and CH0505s-LucR.6ATRi [data not shown]). Of note, while the parental IMCs code for parental Nef proteins, Env-IMC, Env-IMC-LucR.T2A, and Env-IMC-LucR.6ATRi code for the pNL4.3 Nef protein.

### Virus production and infections.

To achieve similar levels of infection among the different IMCs tested, vesicular stomatitis virus G (VSVG)-pseudotyped HIV-1 isolates were produced and titrated as previously described (1). The viruses were then used to infect activated primary CD4 T cells from healthy HIV-1-negative donors by spin infection at 800 × *g* for 1 h in 96-well plates at 25°C. The percentages of infected cells, as evaluated by intracellular p24 staining, were below 15% for all the viruses tested and typically reached 10%.

### Antibodies and sera.

The following Abs were used as primary Abs for cell surface staining: mouse anti-CD4 MAb OKT4 (recognizing the D3 domain of CD4; BioLegend); rabbit anti-BST-2 Ab (sc-99191; Santa Cruz); allophycocyanin (APC)-conjugated mouse anti- MHC-I (clone G46-2.6, recognizing a monomorphic epitope on HLA-A, HLA-B, and HLA-C; BD Biosciences); anti-HIV-1 Env 2G12, 10e8, and A32 MAbs (NIH AIDS Reagent Program); and NKG2D-IgG Fc fusion protein (recognizing NKG2D ligands MICA, MICB, and ULBPs; R&D Systems) or its matched IgG Fc fusion protein (R&D Systems) as a control. Goat anti-mouse and anti-human antibodies precoupled to Alexa Fluor 647 (Invitrogen) were used as secondary antibodies in flow cytometry experiments. Sera from chronically HIV-infected donors were collected, heat inactivated, and conserved as previously described (3, 9). A random number generator (QuickCalcs; GraphPad, San Diego, CA, USA) was used to randomly select a number of sera for each experiment. Antibodies for Western blotting are described below.

### Flow cytometry analysis.

Cell surface staining was performed as previously described (1, 9). Binding of cell surface CD4 (OKT4; 1 μg/ml), MHC-I (clone G46-2.6), and NKG2DL by NKG2D-Fc chimera protein (5 μg/ml) and HIV-1 Env by sera (1:1,000 dilution) or anti-Env MAb A32 (5 μg/ml) was performed at 48 h postinfection. Infected cells were identified by intracellular staining of HIV-1 p24 using a Cytofix/Cytoperm fixation/permeabilization kit (BD Biosciences, Mississauga, ON, Canada) and a fluorescent anti-p24 MAb (phycoerythrin [PE]-conjugated anti-p24, clone KC57; Beckman Coulter/Immunotech). The percentage of infected cells (p24^+^) was determined by gating the live-cell population on the basis of viability dye staining (Aqua Vivid; Thermo Fisher Scientific). Samples were acquired on an LSRII cytometer (BD Biosciences), and data analysis was performed using FlowJo vX.0.7 (Tree Star, Ashland, OR, USA).

### FACS-based ADCC assay.

Measurement of ADCC using a fluorescence-activated cell sorting (FACS)-based assay was performed at 48 h postinfection (hpi) as previously described (3, 9, 82). Briefly, infected primary CD4^+^ T cells were stained with AquaVivid viability dye and cell proliferation dye (eFluor670; eBioscience) and used as target cells. Autologous effector PBMCs, stained with another cellular marker (cell proliferation dye eFluor450; eBioscience), were added at an effector/target ratio of 10:1 in 96-well V-bottom plates (Corning, Corning, NY). A 1:1,000 final dilution of sera or 5 μg/ml of ADCC-mediating MAb was added to appropriate wells, and the cells were incubated for 15 min at room temperature. The plates were subsequently centrifuged for 1 min at 300 × *g* and incubated at 37°C and 5% CO_2_ for 5 to 6 h before being fixed in a 2% phosphate-buffered saline (PBS)-formaldehyde solution. Alternatively, effector cells were preincubated in the presence of purified anti-human CD314 (NKG2D; R&D Systems) or its matched IgG isotype control (10 μg/ml) prior being incubated with target cells for NKG2D blockade experiments. Samples were acquired on an LSRII cytometer (BD Biosciences), and data analysis was performed using FlowJo vX.0.7 (Tree Star). The percentage of ADCC was calculated with the following formula: (percent p24^+^ cells in targets plus effectors) − (percent p24^+^ cells in targets plus effectors plus Abs)/(percent p24^+^ cells in targets) by gating on infected live target cells.

### Western blotting.

CD8-depleted, CD3/CD28 Dynabead (Gibco, Life Technologies)-activated CD4^+^ T cells from 4 healthy non-HIV-infected donors were pooled and infected as described above (with VSV-G-pseudotyped virions). The percentage and total number of infected CD4 T cells were determined at 72 hpi via intracellular p24 staining and flow cytometric analysis (essentially as described above). Aliquots of the cells were lysed with Laemmli sample buffer at a final concentration of 0.5 × 10^4^ infected cells/μl of lysate. Equal amounts (either 10 μl or 20 μl) of lysate from each sample were loaded in parallel for p24 and Nef detection. The procedures for sample sonication, denaturing SDS-PAGE, and Western blotting were essentially as we described previously (35). Here, we used the following primary antibodies: polyclonal rabbit HIV-1 Nef antiserum (obtained from the NIH AIDS Reagent Program, Division of AIDS, NIAID, NIH; contributed by Ronald Swanstrom; catalog no. 2949; lot 10-070932) at 1:1,000 dilution to visualize Nef protein and mouse MAb to HIV-1 p24 (Gag), prepared from the HIV-1 p24 hybridoma (clone 183-H12-5C; obtained from the NIH AIDS Reagent Program, Division of AIDS, NIAID, NIH; contributed by Bruce Chesebro and Hardy Chen; catalog no. 1513) (83) and used at 1:1,000 dilution to probe for p24/Gag.

### Statistical analyses.

Statistics were analyzed using GraphPad Prism version 6.01 (GraphPad, San Diego, CA, USA). Every data set was tested for statistical normality, and the information was used to apply the appropriate (parametric or nonparametric) statistical test. *P* values of <0.05 were considered significant; significance values are indicated as *, *P* < 0.05; **, *P* < 0.01, ***, *P* < 0.001; ****, *P* < 0.0001.
